# Supplementary material for: A human electrophysiological signature of Fragile X pathophysiology is shared in V1 of Fmr1-/y mice
Source: Nat Commun. 2026 Feb 9;17:1497. doi: 10.1038/s41467-026-69243-0 (PMC12891484; doi:10.1038/s41467-026-69243-0)
Supplement: Supplementary file 1 — Supplementary Information [file 41467_2026_69243_MOESM1_ESM.pdf]

Supplementary Figures

A human electrophysiological signature of Fragile X pathophysiology is shared in V1 of *Fmr1*$^{-/y}$ mice

Sara S. Kornfeld-Sylla, Cigdem Gelegen, Jordan E. Norris, Francesca A. Chaloner, Maia Lee, Michael Khela, Maxwell J. Heinrich, Peter S. B. Finnie, Lauren E. Ethridge, Craig A. Erickson, Lauren M. Schmitt, Sam F. Cooke, Carol L. Wilkinson, Mark F. Bear

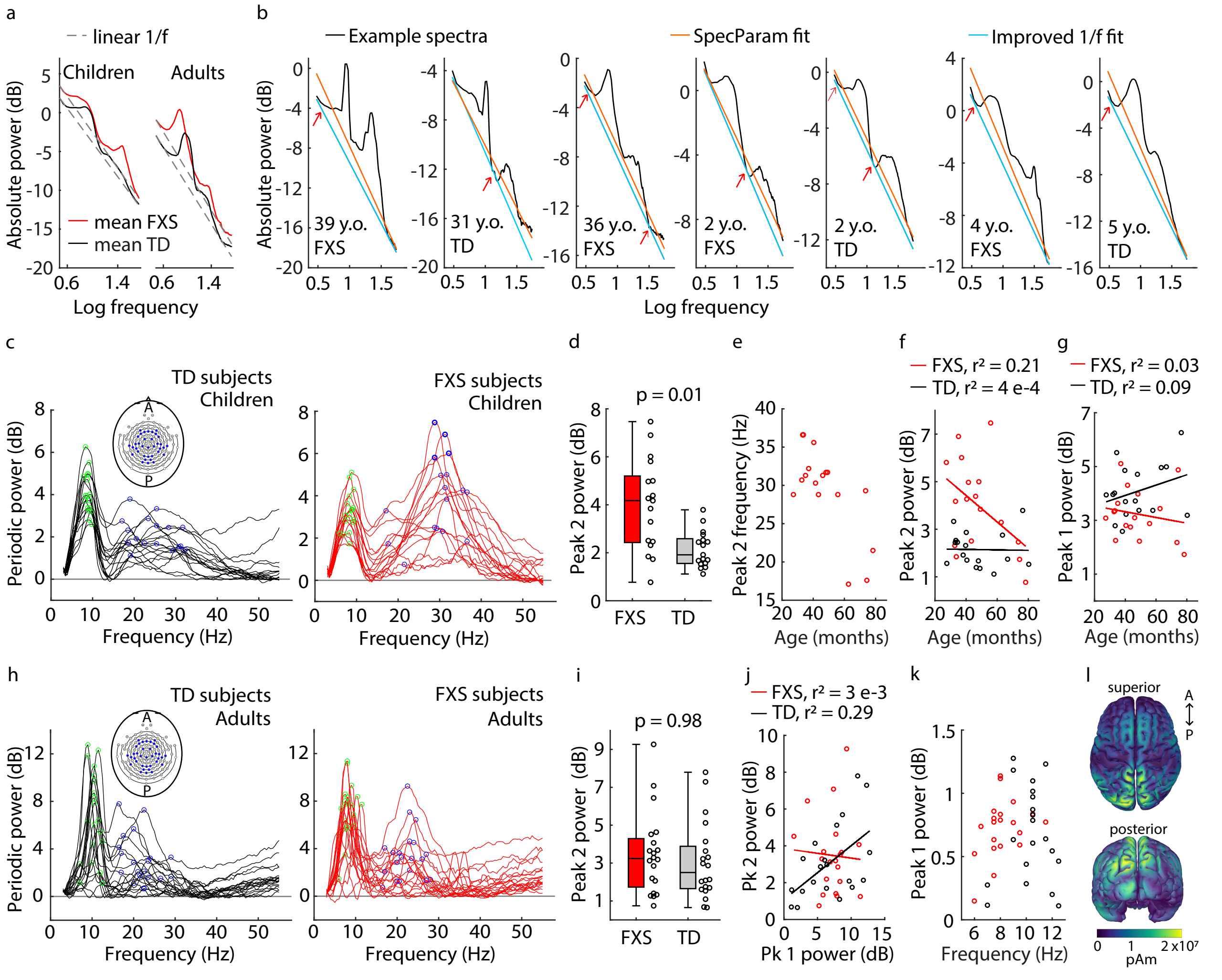

a
linear 1/f
Children
Adults
mean FXS
mean TD
Absolute power (dB)
Log frequency
b
Example spectra
SpecParam fit
Improved 1/f fit
39 y.o. FXS
31 y.o. TD
36 y.o. FXS
2 y.o. FXS
2 y.o. TD
4 y.o. FXS
5 y.o. TD
Absolute power (dB)
Log frequency
c
TD subjects Children
FXS subjects Children
A
P
Periodic power (dB)
Frequency (Hz)
d
p = 0.01
Peak 2 power (dB)
FXS
TD
e
Peak 2 frequency (Hz)
Age (months)
f
FXS, r² = 0.21
TD, r² = 4 e-4
Peak 2 power (dB)
Age (months)
g
FXS, r² = 0.03
TD, r² = 0.09
Peak 1 power (dB)
Age (months)
h
TD subjects Adults
FXS subjects Adults
Periodic power (dB)
Frequency (Hz)
i
p = 0.98
Peak 2 power (dB)
FXS
TD
j
FXS, r² = 3 e-3
TD, r² = 0.29
Pk 2 power (dB)
Pk 1 power (dB)
k
Peak 1 power (dB)
Frequency (Hz)
l
superior
posterior
A
P
0
1
2 x10⁷
pAm

Supplementary Figure 1. 1/f fitting methodology and cross-sectional developmental trajectories of periodic Pk1 and Pk2. a. Average power spectra for rsEEG of both FXS and TD children (n = 17 per group) and adults (n = 20 per group) are best fit by a line in log-log space (linear 1/f). **b.** Methodology. Comparison of the fit generated with the SpecParam code[49] and our improved 1/f fit for spectra from a representative subset of children and adult subjects, plotted in log-log space. Red arrows identify regions where the SpecParam fit overestimates the aperiodic power, which occurred consistently in the 3-6 Hz and 10-20 Hz ranges. Our data-driven approach fits the line to these problematic regions for an improved fit. **c.** Identification of the maximum power and center frequency for each TD (left) and FXS (right) child for periodic Pk1 (green circles) and Pk2 (blue circles). N = 17 subjects per group. A/P = anterior/posterior. **d.** Boxplot and FDR-corrected p-values for Pk2 maximum power in FXS and TD children (uncorrected p-value = 0.002, z-statistic = 3.031, effect size = 0.612). **e.** Pk2 center frequency as a function of age for FXS children. The three outlying values were measured in older subjects. **f-g.** Maximum power as a function of age for Pk2 (f) and Pk1 (g) for FXS children (red circles) and TD children (black circles) with corresponding lines of best fit and r^2 values. For (f), effect size (Pearson's r) = 0.455 (FXS) and 0.021 (TD). For (g), effect size = 0.18 (FXS) and 0.307 (TD). **h-i.** Like (c-d) but for FXS and TD adults. N = 20 subjects per group. For (i), uncorrected p-value = 0.49, z-statistic = 0.69, effect size = 0.13. **j.** Line of best fit and r^2 value for the maximum power of Pk2 as a function of Pk1 maximum power for FXS adults (red circles, effect size = 0.056) and TD adults (black circles, effect size = 0.538). **k.** Pk1 maximum power as a function of Pk1 center frequency for FXS adults (red circles) and TD adults (black circles). **l.** Source localization of mean absolute 4-14 Hz power for adult FXS subjects. pAm = Picoamp x meter. Boxplots in (d,i) show 25th, median, and 75th percentiles, with whiskers extending to minimum and maximum values. Uncorrected p-values and z-statistics calculated from two-sample, two-sided Wilcoxon Rank-Sum Tests and effect sizes calculated using Cliff's Delta (d,i) or Pearson's r (f,g,j). P-values adjusted with the Benjamini-Hochberg correction.

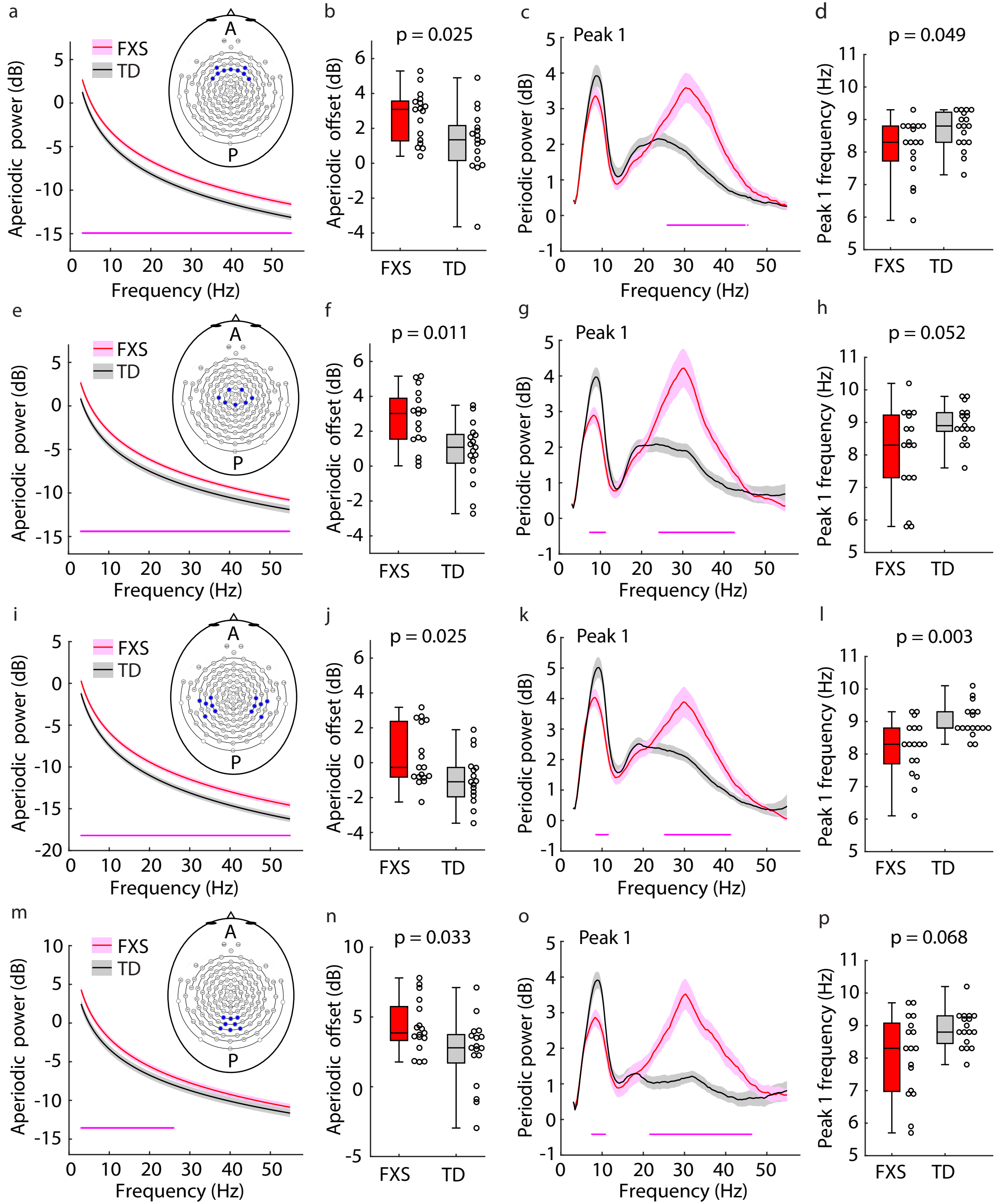
a
FXS
TD
A
P
Aperiodic power (dB)
Frequency (Hz)
b
p = 0.025
Aperiodic offset (dB)
FXS
TD
c
Peak 1
Periodic power (dB)
Frequency (Hz)
d
p = 0.049
Peak 1 frequency (Hz)
FXS
TD
e
FXS
TD
A
P
Aperiodic power (dB)
Frequency (Hz)
f
p = 0.011
Aperiodic offset (dB)
FXS
TD
g
Peak 1
Periodic power (dB)
Frequency (Hz)
h
p = 0.052
Peak 1 frequency (Hz)
FXS
TD
i
FXS
TD
A
P
Aperiodic power (dB)
Frequency (Hz)
j
p = 0.025
Aperiodic offset (dB)
FXS
TD
k
Peak 1
Periodic power (dB)
Frequency (Hz)
l
p = 0.003
Peak 1 frequency (Hz)
FXS
TD
m
FXS
TD
A
P
Aperiodic power (dB)
Frequency (Hz)
n
p = 0.033
Aperiodic offset (dB)
FXS
TD
o
Peak 1
Periodic power (dB)
Frequency (Hz)
p
p = 0.068
Peak 1 frequency (Hz)
FXS
TD

Supplementary Figure 2. rsEEG phenotypes of FXS children by region of interest (ROI). a. Aperiodic fit (mean +/- SEM) for electrodes in the frontal ROI for FXS and TD children (n = 17 per group). **b.** Boxplot and p-value for aperiodic offset (the power at 3 Hz) from frontal electrodes. Z-statistic = 2.239, effect size = 0.453. **c.** Periodic power (mean +/- SEM) for frontal electrodes. **d.** Boxplot and p-value for Pk1 center frequency from frontal electrodes. Z-statistic = -1.971, effect size = -0.394. **f-p.** Like (a-d) but for electrodes in the central (e-h), temporal (i-l) and occipital (m-p) ROIs. For (f), z-statistic = 2.549 and effect size = 0.516. For (h), z-statistic = -1.942 and effect size = -0.391. For (j), z-statistic = 2.239 and effect size = 0.453. For (l), z-statistic = -2.949 and effect size = -0.585. For (n), z-statistic = 2.136 and effect size = 0.433. For (p), z-statistic = -1.824 and effect size = -0.367. Dots at bottom of plots in (a,c,e,g,i,k,m,o) indicate the points of significant difference between groups (non-parametric hierarchical bootstrap, 95% confidence interval). Boxplots show 25th, median, and 75th percentiles, with whiskers extending to minimum and maximum values. P-values and z-statistics calculated from two-sample, two-sided Wilcoxon Rank-Sum Tests (no multiple comparisons) and effect sizes calculated using Cliff's Delta.

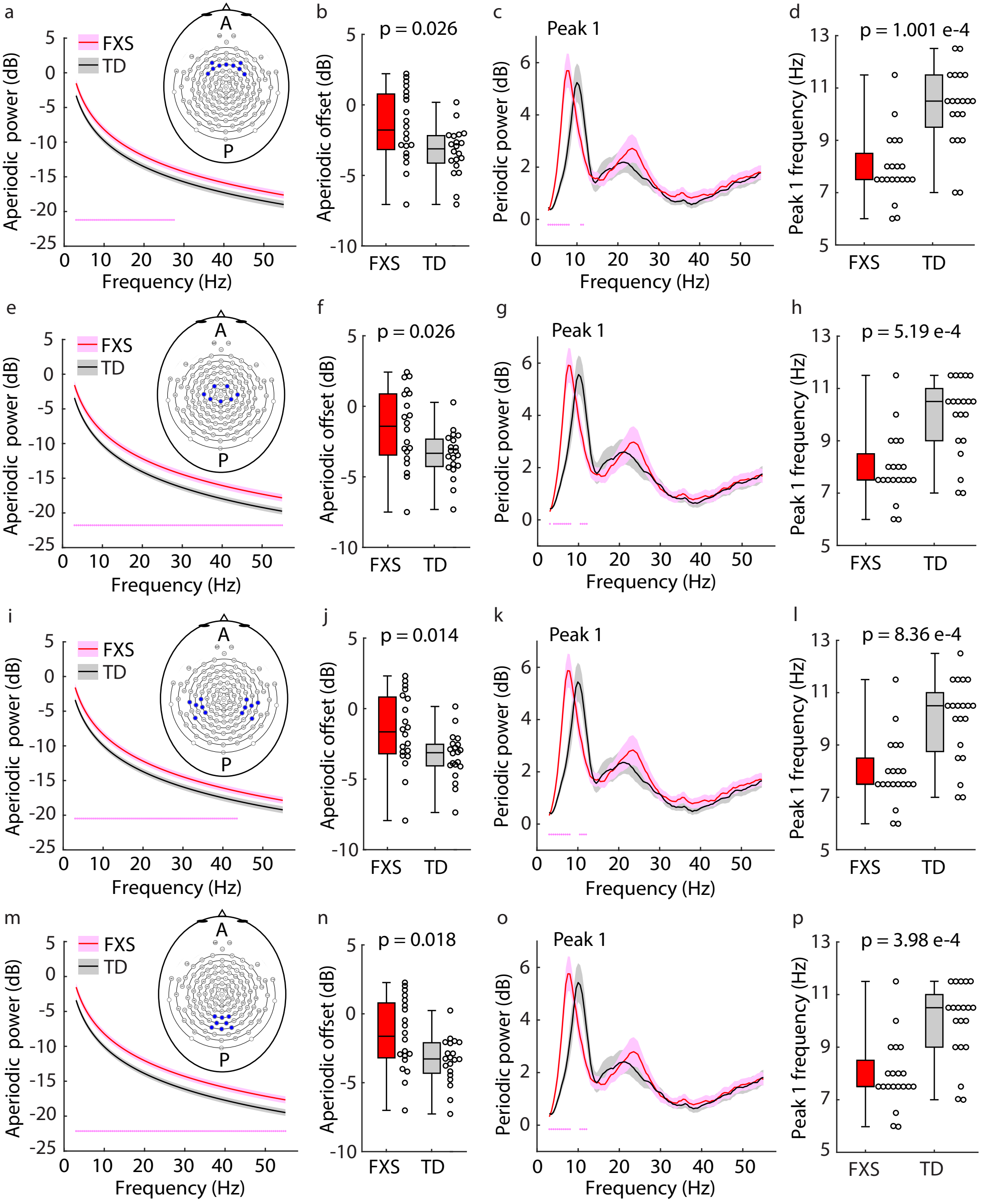
a
FXS
TD
A
P
Aperiodic power (dB)
Frequency (Hz)
b
p = 0.026
Aperiodic offset (dB)
FXS
TD
c
Peak 1
Periodic power (dB)
Frequency (Hz)
d
p = 1.001 e-4
Peak 1 frequency (Hz)
FXS
TD
e
FXS
TD
A
P
Aperiodic power (dB)
Frequency (Hz)
f
p = 0.026
Aperiodic offset (dB)
FXS
TD
g
Peak 1
Periodic power (dB)
Frequency (Hz)
h
p = 5.19 e-4
Peak 1 frequency (Hz)
FXS
TD
i
FXS
TD
A
P
Aperiodic power (dB)
Frequency (Hz)
j
p = 0.014
Aperiodic offset (dB)
FXS
TD
k
Peak 1
Periodic power (dB)
Frequency (Hz)
l
p = 8.36 e-4
Peak 1 frequency (Hz)
FXS
TD
m
FXS
TD
A
P
Aperiodic power (dB)
Frequency (Hz)
n
p = 0.018
Aperiodic offset (dB)
FXS
TD
o
Peak 1
Periodic power (dB)
Frequency (Hz)
p
p = 3.98 e-4
Peak 1 frequency (Hz)
FXS
TD

Supplementary Figure 3. rsEEG phenotypes of FXS adults by region of interest (ROI). a-p. Like Supplementary Fig. 2 but for FXS and TD adults (n = 20 per group). For (b), z-statistic = 2.232 and effect size = 0.415. For (d), z-statistic = -3.89 and effect size = -0.715. For (f), z-statistic = 2.232 and effect size = 0.415. For (h), z-statistic = -3.471 and effect size = -0.638. For (j), z-statistic = 2.245 and effect size = 0.455. For (l), z-statistic = -3.341 and effect size = -0.615. For (n), z-statistic = 2.367 and effect size = 0.44. For (p), z-statistic = -3.541 and effect size = -0.65. Dots at bottom of plots in (a,c,e,g,i,k,m,o) indicate the points of significant difference between groups (non-parametric hierarchical bootstrap, 95% confidence interval). Boxplots show 25th, median, and 75th percentiles, with whiskers extending to minimum and maximum values. Uncorrected p-values and z-statistics calculated from two-sample, two-sided Wilcoxon Rank-Sum Tests (no multiple comparisons) and effect sizes calculated using Cliff's Delta.

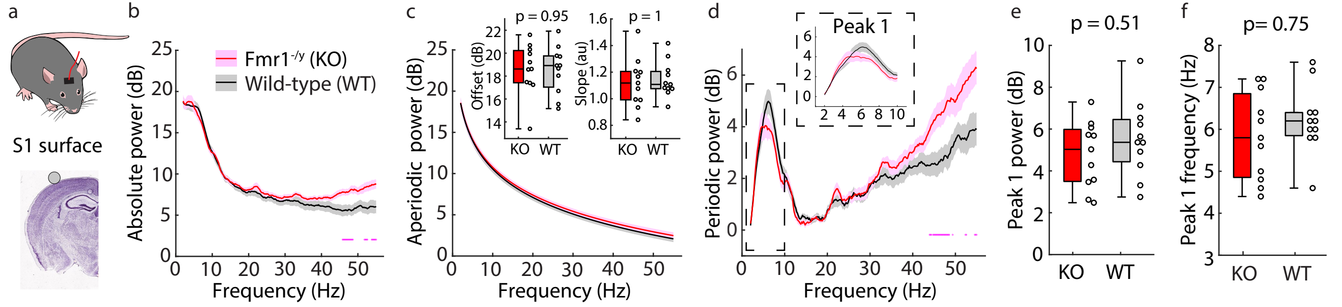

a
S1 surface
b
Fmr1-/y (KO)
Wild-type (WT)
Absolute power (dB)
Frequency (Hz)
c
Aperiodic power (dB)
Offset (dB)
p = 0.95
Slope (au)
p = 1
KO
WT
Frequency (Hz)
d
Periodic power (dB)
Peak 1
Frequency (Hz)
e
p = 0.51
Peak 1 power (dB)
KO
WT
f
p= 0.75
Peak 1 frequency (Hz)
KO
WT

Supplementary Figure 4. Pk1 center frequency is more variable in S1 of adult *Fmr1*$^{-/y}$ mice. **a.** Experimental design. EEG data were collected from an electrode on the cortical surface of S1 of freely-moving adult *Fmr1*$^{-/y}$ (KO) and littermate WT mice (p70-115, n = 11 per group). 100 sec of resting-state data while the mice were awake but stationary were analyzed. Nissl from the Allen Reference Atlas – Mouse Brain[119], https://atlas.brain-map.org/. **b.** Absolute power spectrum (mean +/- SEM). **c.** Main: Aperiodic fit (mean +/- SEM). Inset: Boxplots and FDR-corrected p-values for aperiodic offset (power at 2 Hz) and aperiodic slope. Offset z-statistic = 0.066, effect size = 0.0248; slope uncorrected p-value = 0.793, z-statistic = -0.263, effect size = -0.074. **d.** Periodic power (mean +/- SEM). Power in KO mice is significantly elevated between 44.8-55 Hz. Inset shows zoom on periodic peak (Pk1). **e-f.** Boxplot and FDR-corrected p-values for: (e) Pk1 maximum power. Z-statistic = -0.657, effect size = -0.174; and (f) Pk1 center frequency. Uncorrected p-value = 0.373, z-statistic = -0.89, effect size = -0.231. Dots at bottom of plots in (b,d) indicate points of significant difference between groups (non-parametric hierarchical bootstrap, 99% confidence interval). Boxplots show 25th, median, and 75th percentiles, with whiskers extending to minimum and maximum values. Uncorrected p-values and z-statistics calculated from two-sample, two-sided Wilcoxon Rank-Sum Tests and effect sizes calculated using Cliff's Delta. P-values adjusted with the Benjamini-Hochberg correction. Source data are provided as a Source Data file.

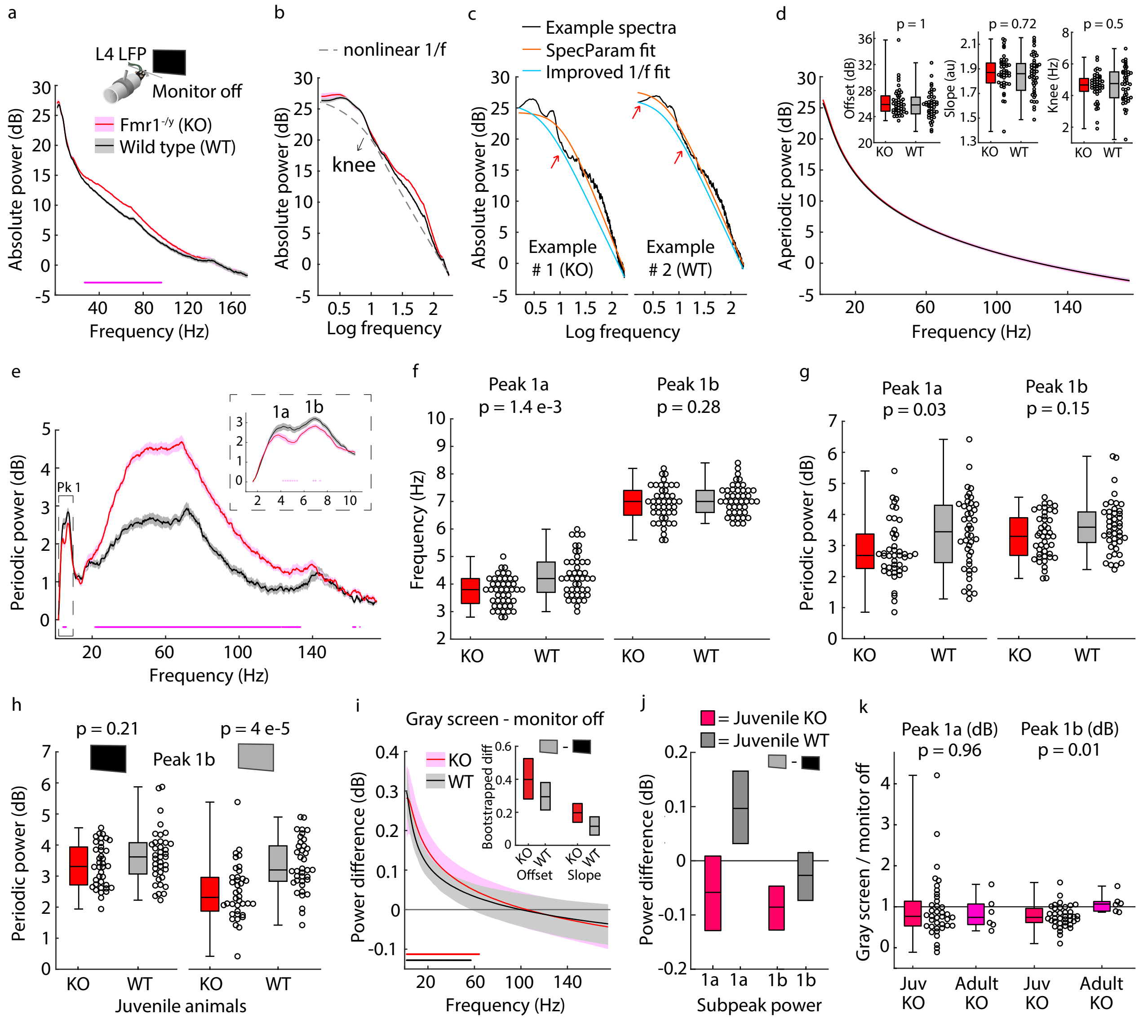

a
L4 LFP
Monitor off
Fmr1-/y (KO)
Wild type (WT)
Absolute power (dB)
Frequency (Hz)
b
nonlinear 1/f
knee
Absolute power (dB)
Log frequency
c
Example spectra
SpecParam fit
Improved 1/f fit
Example # 1 (KO)
Example # 2 (WT)
Absolute power (dB)
Log frequency
d
p = 1
p = 0.72
p = 0.5
Offset (dB)
Slope (au)
Knee (Hz)
KO WT
Aperiodic power (dB)
Frequency (Hz)
e
Pk 1
1a 1b
Periodic power (dB)
Frequency (Hz)
f
Peak 1a
p = 1.4 e-3
Peak 1b
p = 0.28
Frequency (Hz)
KO WT KO WT
g
Peak 1a
p = 0.03
Peak 1b
p = 0.15
Periodic power (dB)
KO WT KO WT
h
p = 0.21
p = 4 e-5
Peak 1b
Periodic power (dB)
KO WT KO WT
Juvenile animals
i
Gray screen - monitor off
KO
WT
Bootstrapped diff
Offset Slope
Power difference (dB)
Frequency (Hz)
j
= Juvenile KO
= Juvenile WT
Power difference (dB)
1a 1a 1b 1b
Subpeak power
k
Peak 1a (dB)
p = 0.96
Peak 1b (dB)
p = 0.01
Gray screen / monitor off
Juv KO
Adult KO
Juv KO
Adult KO

Supplementary Figure 5. Luminance affects genotype differences in periodic Pk1 power. a. Inset**:** Experimental design. V1 L4 LFP data were collected in *Fmr1*$^{-/y}$ (KO) and littermate WT mice (n = 44 per group, p30-150) head-fixed in front of a monitor that was turned off (i.e., mice were in the dark) after two days of habituation to head-fixation. Main: Absolute power spectrum (mean +/- SEM) from 150 sec of data. **b.** Methodology. Like (a) but plotted in log-log space, revealing the nonlinear 1/f. The inflection point of the curve is captured in the knee frequency parameter. **c.** Comparison of the fit generated with the SpecParam code[49] and our improved 1/f fit for spectra from an example KO and WT mouse, plotted in log-log space. Red arrows identify regions where the SpecParam fit overestimates the aperiodic power, which occurred consistently in the 1.5-4 Hz and 10-20 Hz ranges, similar to the EEG data. Our data-driven approach fits to the problematic 10-20 Hz region for an improved fit. **d.** Main: Aperiodic fit (mean +/- SEM) of power spectra in (a). Inset: Boxplot and FDR-corrected p-values for aperiodic offset (power at 1.5 Hz), slope and knee frequency. For offset, uncorrected p-value = 0.339, z-statistic = 0.956, effect size = 0.119. For slope, uncorrected p-value = 0.481, z-statistic = 0.705, effect size = 0.088. For knee frequency, z-statistic = -0.672, effect size = -0.084. **e.** Main: Periodic power (mean +/- SEM). Dots at bottom of plots in (a,e) indicate the points of significant difference between groups (non-parametric hierarchical bootstrap, 99% confidence interval (CI)). Inset: Higher spectral resolution reveals Pk1 has two sub-peaks. **f.** Boxplot and FDR-corrected p-values for Pk1a and Pk1b center frequency. For Pk1a, uncorrected p-value = 3.6e-4, z-statistic = -3.57, effect size = -0.441. For Pk1b, z-statistic = -1.087, effect size = -0.134. **g.** Like (f) but for Pk1a and Pk1b maximum power. For Pk1a, uncorrected p-value = 0.016, z-statistic = -2.408, effect size = -0.299. For Pk1b, uncorrected p-value = 0.114, z-statistic = -1.581, effect size = -0.196. **h.** Pk1b maximum power for juvenile (p30-40) KO and WT mice (n = 38 per group) during 'monitor off' (z-statistic = -1.252, effect size = -0.166) and 'gray screen' (z-statistic = -4.101, effect size = -0.549). **i.** Main: Bootstrapped difference (median +/- 99% CI) between aperiodic fits for 'gray screen' and 'monitor off' for all KO and WT mice (n = 44 per group). Dots at the bottom indicate points of significant difference between 'gray screen' and 'monitor off' (red dots for KO, black dots for WT). Inset: Bootstrapped difference (median +/- 99% CI) between 'gray screen' and 'monitor off' for aperiodic offset (units dB) and slope (units au). **j.** Bootstrapped difference (median +/- 99% CI) between 'gray screen' and 'monitor off' for the maximum power of Pk1a and Pk1b for juvenile (p30-40) mice (n = 38 per group). **k.** Boxplot and FDR-corrected p-values for the ratio of 'monitor off' and 'gray screen' periodic Pk1a (left) and Pk1b (right) maximum power for juvenile (n = 38) and adult (n = 6) KO mice. For Pk1a, z-statistic = -0.051, effect size = -0.018. For Pk1b, uncorrected p-value = 0.0048, z-statistic = -2.821, effect size = -0.728. Boxplots show 25th, median, and 75th percentiles, with whiskers extending to minimum and maximum values. Uncorrected p-values and z-statistics calculated from two-sample, two-sided Wilcoxon Rank-Sum Tests and effect sizes calculated using Cliff's Delta. P-values adjusted with the Benjamini-Hochberg correction. Source data are provided as a Source Data file.

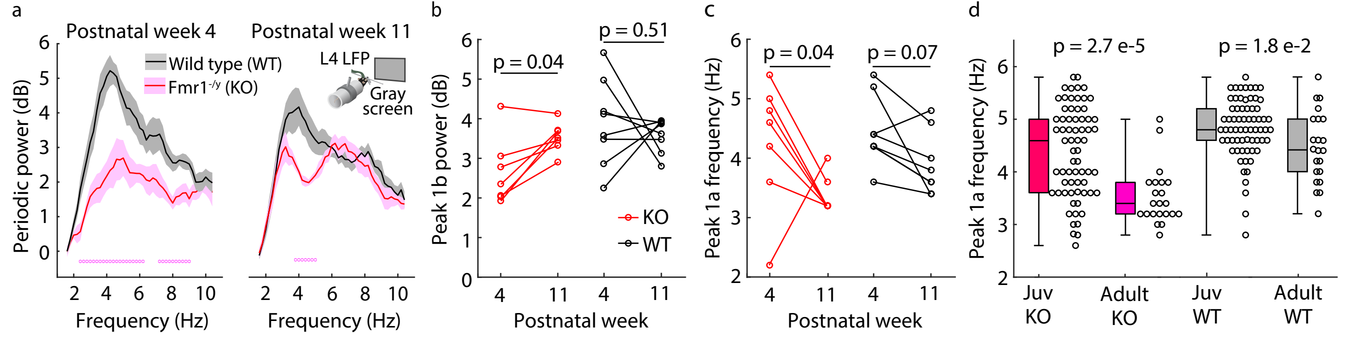

a
Postnatal week 4
Postnatal week 11
Wild type (WT)
Fmr1-/y (KO)
L4 LFP
Gray screen
Periodic power (dB)
Frequency (Hz)
b
p = 0.04
p = 0.51
Peak 1b power (dB)
KO
WT
Postnatal week
c
p = 0.04
p = 0.07
Peak 1a frequency (Hz)
Postnatal week
d
p = 2.7 e-5
p = 1.8 e-2
Peak 1a frequency (Hz)
Juv KO
Adult KO
Juv WT
Adult WT

Supplementary Figure 6. Longitudinal developmental trajectory for periodic Pk1 phenotypes supports cross-sectional findings. a. Inset**:** Experimental design. V1 L4 LFP data were collected in head-fixed *Fmr1*$^{-/y}$ (KO) mice (n = 7) and littermate WT mice (n = 8) viewing a static gray screen at two different time points: postnatal week 4 (p28-34, left) and week 11 (p77-83, right). Main: Periodic Pk1 spectrum (mean +/- SEM) from 120 sec of LFP data at each timepoint. Dots at bottom of the plots indicate the points of significant difference between groups at each timepoint (non-parametric hierarchical bootstrap, 99% confidence interval). **b.** Pk1b maximum power for each genotype at each timepoint. Due to potential electrode drift and other confounds of longitudinal recordings, datasets were conservatively treated as independent. P-values from comparing across timepoints within a genotype. Z-statistics and effect sizes are not calculated for n < 10. **c.** Like (b), but for the center frequency of Pk1a. **d.** Boxplot and p-values for the center frequency of Pk1a for juvenile (p30-40, n = 67) and adult (p70-150, n = 22) KO (left) and WT (right) mice viewing the static gray screen from the cross-sectional study. For KO mice, z-statistic = 4.199 and effect size = 0.598. For WT mice, z-statistic = 2.369 and effect size = 0.337. P-values in (b-d) and z-statistics in (d) calculated from two-sample, two-sided Wilcoxon Rank-Sum Tests (no multiple comparisons). Effect sizes in (d) calculated using Cliff's Delta. Source data are provided as a Source Data file.

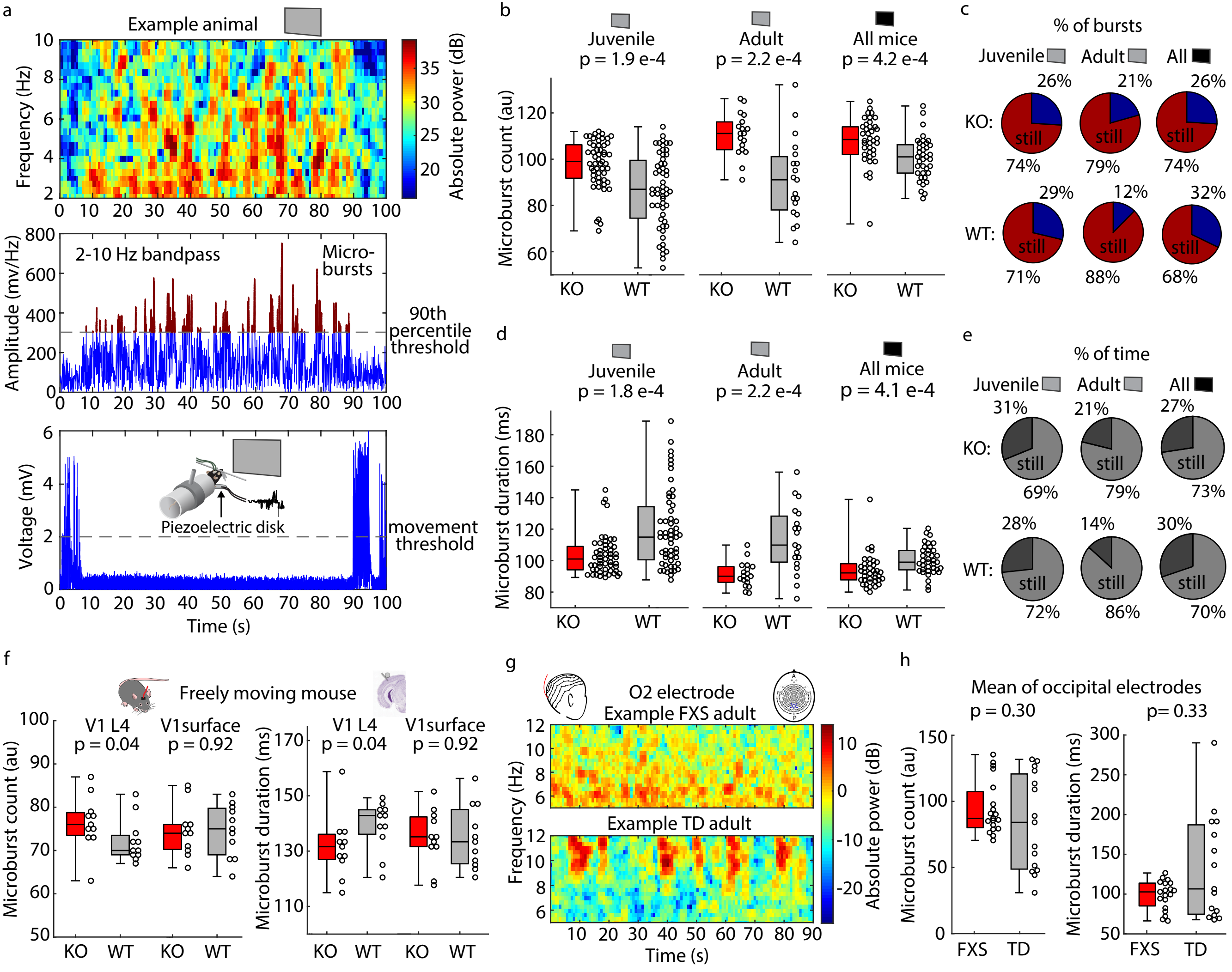
a
Example animal
Frequency (Hz)
Absolute power (dB)
2-10 Hz bandpass
Micro-bursts
90th percentile threshold
Amplitude (mv/Hz)
Piezoelectric disk
movement threshold
Voltage (mV)
Time (s)
b
Juvenile
p = 1.9 e-4
Adult
p = 2.2 e-4
All mice
p = 4.2 e-4
Microburst count (au)
KO
WT
c
% of bursts
Juvenile
Adult
All
KO:
WT:
still
26%
74%
21%
79%
26%
74%
29%
71%
12%
88%
32%
68%
d
Juvenile
p = 1.8 e-4
Adult
p = 2.2 e-4
All mice
p = 4.1 e-4
Microburst duration (ms)
e
% of time
31%
69%
21%
79%
27%
73%
28%
72%
14%
86%
30%
70%
f
Freely moving mouse
V1 L4
p = 0.04
V1surface
p = 0.92
Microburst count (au)
Microburst duration (ms)
g
O2 electrode
Example FXS adult
Example TD adult
Frequency (Hz)
Absolute power (dB)
Time (s)
h
Mean of occipital electrodes
p = 0.30
p= 0.33
Microburst count (au)
Microburst duration (ms)
FXS
TD

Supplementary Figure 7. Alpha-like oscillations in V1 of *Fmr1*$^{-/y}$ mice exhibit altered temporal dynamics not detectable from the surface. a. Methodology. Time series from an example animal viewing a static gray screen. Across 100 sec of continuous data, power between 2-10 Hz fluctuates in a burst-like pattern. Band-passing filtering and applying a Hilbert transform to the 2-10 Hz signal revealed the number of times the signal crosses the 90th percentile amplitude value (microburst count) and the length of time the signal stays above the 90th percentile threshold after each crossing (microburst duration). Timing of bursts was compared to timing of movement bouts, measured through a piezoelectric disk under the animal's forepaw. Altered pressure on the disk during movement was recorded as a voltage deflection, and rectified voltages values above 2 mV constituted movement. **b.** Boxplot and p-values for the microburst count for juvenile (left, p30-40, n = 53 KO and n = 55 WT) and adult (middle, p70-150, n = 18 KO and n = 19 WT) mice viewing gray screen, and for all mice during 'monitor off' (right, n = 38 KO and n = 37 WT). Z-statistic = 3.738 and effect size = 0.418 (juvenile gray screen), z-statistic = 3.694 and effect size = 0.713 (adult gray screen), and z-statistic = 3.526 and effect size = 0.474 ('monitor off') **c.** Distribution of average burst activity during moving and still states across KO (top) and WT (bottom) animals viewing gray screen divided by juveniles (left) and adults (middle) and for all animals during 'monitor off' (right). **d.** Like (b) but for microburst duration. Z-statistic = -3.75 and effect size = -0.419 (juvenile gray screen), z-statistic = -3.693 and effect size = -0.713 (adult gray screen), and z-statistic = -3.537 and effect size = -0.475 ('monitor off'). **e.** Percentage of time spent moving and still across all KO (top) and WT (bottom) animals viewing gray screen divided by juveniles (left, n = 67 per group) and adults (middle, n = 22 per group), and all animals during 'monitor off' (right, n = 44 per group). **f.** Box plots and p-values comparing burst dynamics in freely-moving KO and WT adult mice (n = 11 per group) with electrodes on the surface of V1 and implanted in L4 in the same hemisphere. Nissl from the Allen Reference Atlas – Mouse Brain[119], https://atlas.brain-map.org/. Significant differences in microburst count (left) and duration (right) between groups were only present in L4. For the L4 electrode, z-statistic = -2.039 for microburst count and 2.039 for duration and effect sizes = 0.521 for count and -0.521 for duration. For surface electrode, z-statistic = +/-0.099, effect size +/-0.033 for burst count and duration. **g.** Spectral dynamics across 90 sec of continuous data from the O2 occipital electrode in an example FXS (top) and TD (bottom) adult human subject. Burst activity is present in both subjects and centered around their respective periodic Pk1 maximum values (5.5-9.5 Hz for FXS, 9-12 Hz TD). **h.** Boxplot and p-values for mean microburst count (left) and duration (right) across all electrodes in the occipital ROI for FXS (n = 18) and TD (n = 15) adult subjects. Z-statistic = 1.041 and effect size = 0.214 for counts, and z-statistic = -0.971 and effect size = -0.2 for durations. The variance of the TD distribution was significantly higher (p = 0.0018 for microburst count, p = 2.59e-5 for burst duration, Levene tests). Boxplots show 25th, median, and 75th percentiles, with whiskers extending to minimum and maximum values. P-values and z-statistics calculated from two-sample, two-sided Wilcoxon Rank-Sum Tests (no multiple comparisons) and effect sizes calculated using Cliff's Delta. Source data are provided as a Source Data file.

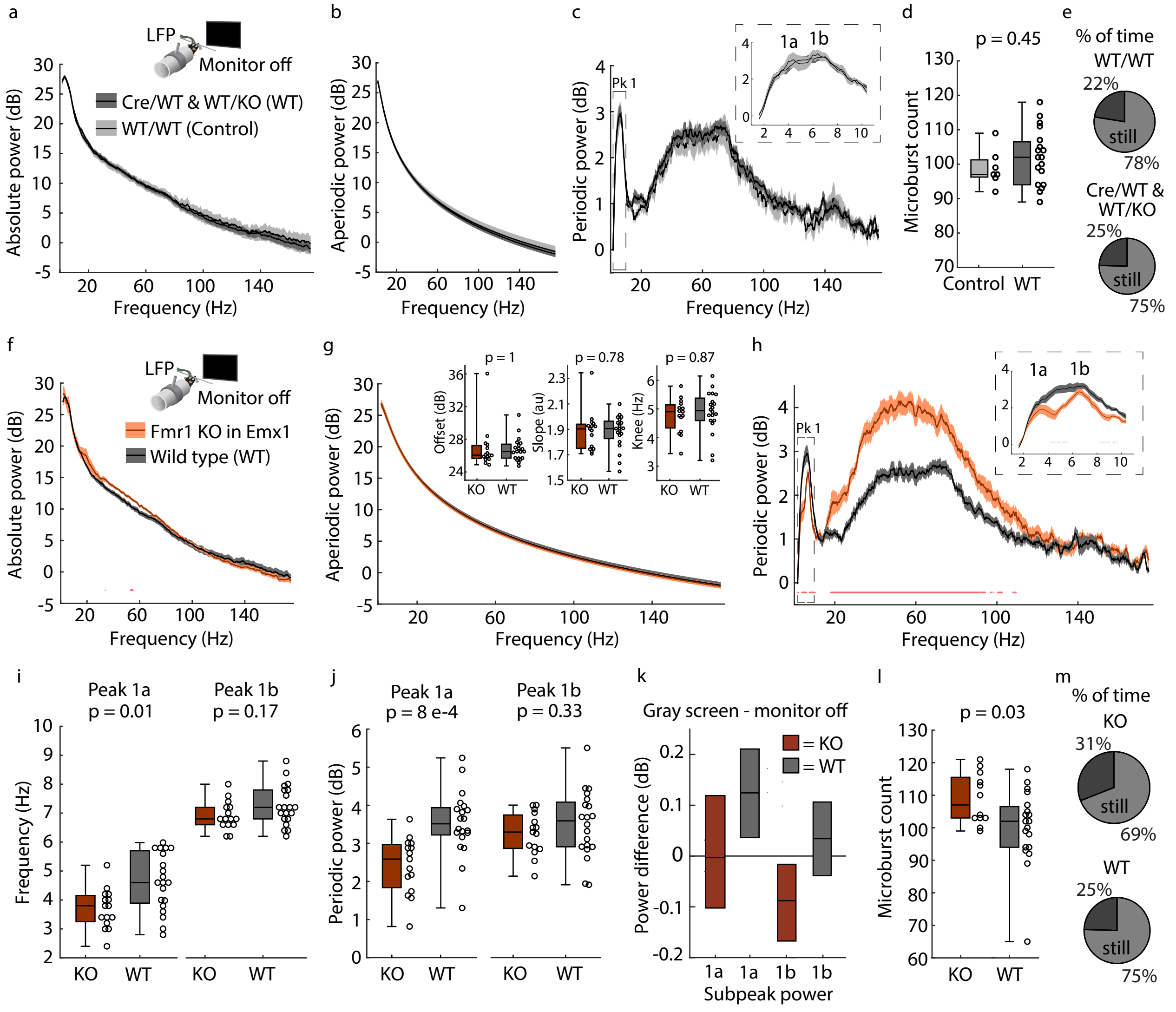

a
LFP
Monitor off
Cre/WT & WT/KO (WT)
WT/WT (Control)
Absolute power (dB)
Frequency (Hz)
b
Aperiodic power (dB)
Frequency (Hz)
c
Pk 1
1a
1b
Periodic power (dB)
Frequency (Hz)
d
p = 0.45
Microburst count
Control
WT
e
% of time
WT/WT
22%
still
78%
Cre/WT & WT/KO
25%
still
75%
f
LFP
Monitor off
Fmr1 KO in Emx1
Wild type (WT)
Absolute power (dB)
Frequency (Hz)
g
p = 1
p = 0.78
p = 0.87
Offset (dB)
Slope (au)
Knee (Hz)
KO
WT
Aperiodic power (dB)
Frequency (Hz)
h
Pk 1
1a
1b
Periodic power (dB)
Frequency (Hz)
i
Peak 1a
p = 0.01
Peak 1b
p = 0.17
Frequency (Hz)
KO
WT
j
Peak 1a
p = 8 e-4
Peak 1b
p = 0.33
Periodic power (dB)
KO
WT
k
Gray screen - monitor off
= KO
= WT
Power difference (dB)
1a
1b
Subpeak power
l
p = 0.03
Microburst count
KO
WT
m
% of time
KO
31%
still
69%
WT
25%
still
75%

Supplementary Figure 8. WT mice in the triple-transgenic experiment match expected control values and Emx1-*Fmr1* KO mice replicate all phenotypes during 'monitor off.' a. Absolute power spectrum (mean +/- SEM) from the L4 LFP electrode in V1 of juvenile (p30-40) wild-type mice in the dark (monitor off) from the triple-transgenic Emx1-*Fmr1* KO sub-strain. Littermate WT/WT (control) mice (n = 9) are compared to Cre/WT and WT/KO mice (WT used in Fig. 4, combined n = 20 mice). **b.** Aperiodic fit (mean +/- SEM). **c.** Periodic spectra (mean +/- SEM). **d.** Boxplot and p-value for microburst count. Z-statistic = -0.753 and effect size = -0.203. n = 7 control, n = 19 WT. **e.** Average percentage of time spent moving and still across 100 sec in all WT/WT (top, n = 9) and all combined Cre/WT and WT/KO mice (bottom, n = 20). **f.** Inset: Experimental design. V1 L4 LFP data were collected from juvenile (p30-40) mice with *Fmr1* specifically knocked out of cortical excitatory neurons and glia expressing the Emx1 promotor (Emx1-*Fmr1* KO; Cre+/Fmr1-, n = 15) and WT littermates (n = 20 in total, combination of Cre+/Fmr1+; Cre/WT, n = 10 and Cre-/Fmr1-; WT/KO, n = 10). Mice were head-fixed in front of a monitor and habituated to head-fixation for two days before data collection. Main: Absolute power spectrum (mean +/- SEM). **g.** Aperiodic fit (mean +/- SEM). Inset: Boxplot and FDR-corrected p-values for aperiodic offset (power at 1.5 Hz), slope and knee frequency. For offset, uncorrected p-value = 0.56, z-statistic = -0.583, effect size = -0.012. For slope, z-statistic = -0.283, effect size = -0.06. For knee, uncorrected p-value = 0.582, z-statistic = -0.55, effect size = -0.011. **h.** Periodic power spectrum (mean +/- SEM). **i-j.** Boxplot and FDR-corrected p-values for the center frequency (i) and maximum power (j) of Pk1a and Pk1b. In (i), Pk1a uncorrected p-value = 0.007, z-statistic = -2.69, and effect size = -0.54. Pk1b uncorrected p-value = 0.127, z-statistic = -1.527, and effect size = -0.307. In (j), Pk1a uncorrected p-value = 2e-4, z-statistic = -3.717, and effect size = -0.747. Pk1b z-statistic = -0.983 and effect size = -0.2. **k.** Bootstrapped difference (median +/- 99% confidence interval) between gray screen and black screen for the maximum power of Pk1a and Pk1b. **l.** Boxplot and p-value for microburst count. Z-statistic = 2.112 and effect size = 0.461. n = 12 KO, n = 19 WT. **m.** Percentage of time spent moving and still across all Emx1-*Fmr1* KO mice (top, n = 15) and WT mice (bottom, n = 20). Dots at bottom of plots in (f,h) indicate points of significant difference between groups (non-parametric hierarchical bootstrap, 99% confidence interval). Boxplots show 25th, median, and 75th percentiles, with whiskers extending to minimum and maximum values. Uncorrected p-values and z-statistics calculated from two-sample, two-sided Wilcoxon Rank-Sum Tests and effect sizes calculated using Cliff's Delta. P-values adjusted with the Benjamini-Hochberg correction. Source data are provided as a Source Data file.

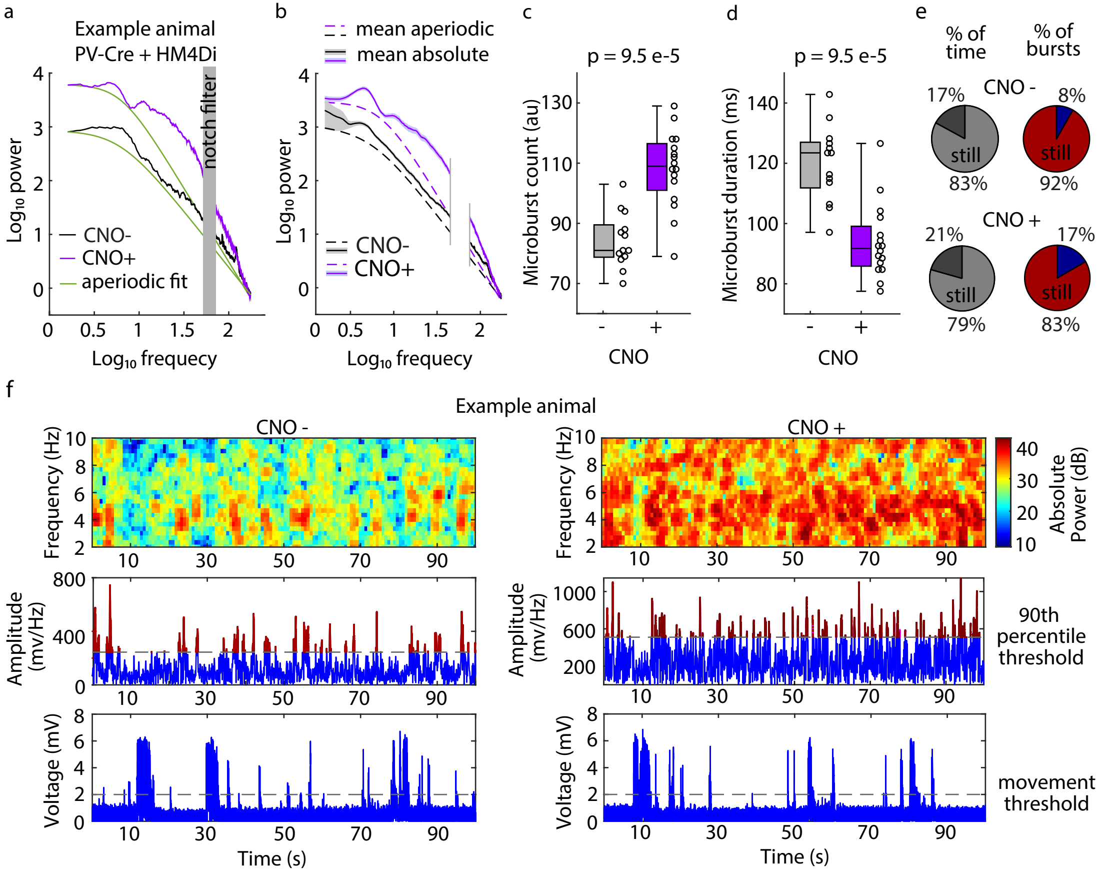

a
Example animal
PV-Cre + HM4Di
notch filter
Log10 power
Log10 frequecy
CNO-
CNO+
aperiodic fit
b
mean aperiodic
mean absolute
Log10 power
Log10 frequecy
CNO-
CNO+
c
p = 9.5 e-5
Microburst count (au)
CNO
d
p = 9.5 e-5
Microburst duration (ms)
CNO
e
% of time
% of bursts
CNO -
17%
83%
8%
92%
still
CNO +
21%
79%
17%
83%
still
f
Example animal
CNO -
CNO +
Frequency (Hz)
Absolute Power (dB)
Amplitude (mv/Hz)
90th percentile threshold
Voltage (mV)
movement threshold
Time (s)

Supplementary Figure 9. Large aperiodic changes from inactivating V1 PV+ interneurons. **a.** Fit for the aperiodic component from an example PV-Cre animal with the chemogenetic actuator hM4Di expressed in V1 before (black) and after (purple) CNO systemic injection. The fitting is done in log-log space and is unaffected by the notch-filtered frequencies (45-75 Hz) due to our curve-fitting algorithm. **b.** Absolute power spectra (mean +/- SEM) for all PV-Cre mice (n = 16) plotted in log-log space. The mean aperiodic fit before (black) and after (purple) CNO injection is plotted below each corresponding absolute spectrum. **c-d.** Boxplot and p-values for microburst count (c) and mean duration (d). Z-statistic = -3.903 (count) and 3.903 (duration), and effect size = -0.8802 (count) and 0.8802 (duration). n = 13 (before CNO) and n = 16 (after CNO). **e.** Left: distribution of average microburst activity during moving and still states across 100 sec for all PV-Cre mice before (top, n = 16) and after (bottom, n = 16) systemic CNO injection. Right: Mean percentage of time spent moving and still across all PV-Cre mice before (top) and after (bottom) CNO injection. **f.** Time series, 2-10 Hz band-passed and Hilbert-transformed signal, and piezoelectric voltage signal for an example PV-Cre+HM4di animal before (left) and after (right) CNO treatment. The increased background (aperiodic) low frequency power affects the burst metrics. Boxplots show 25th, median, and 75th percentiles, with whiskers extending to minimum and maximum values. P-values and z-statistics calculated from two-sample, two-sided Wilcoxon Signed-Rank Tests (no multiple comparisons). Effect sizes calculated using Cliff's Delta. Source data are provided as a Source Data file.

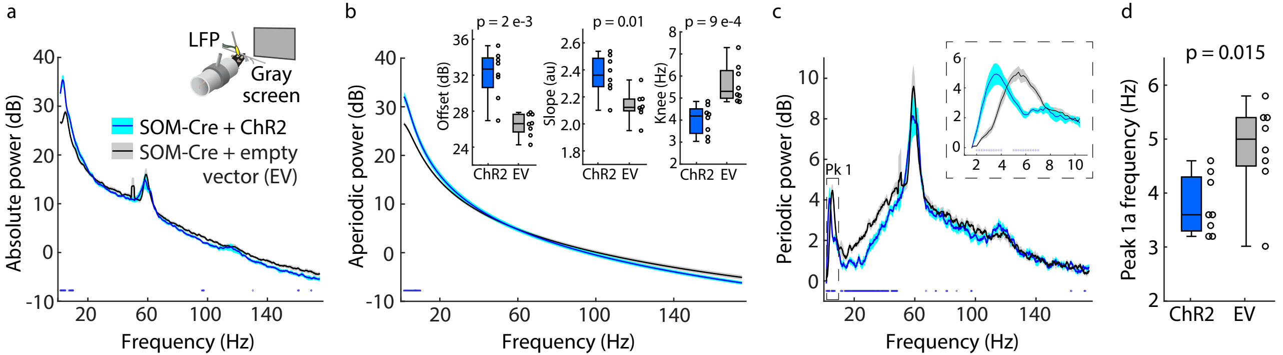
a
LFP
Gray screen
SOM-Cre + ChR2
SOM-Cre + empty vector (EV)
Absolute power (dB)
Frequency (Hz)
b
p = 2 e-3
p = 0.01
p = 9 e-4
Offset (dB)
Slope (au)
Knee (Hz)
ChR2 EV
Aperiodic power (dB)
Frequency (Hz)
c
Pk 1
Periodic power (dB)
Frequency (Hz)
d
p = 0.015
Peak 1a frequency (Hz)
ChR2
EV

Supplementary Figure 10. Optogenetically activating SOM+ interneurons reduces Pk1a center frequency. a. Inset: V1 L4 LFP data were collected in SOM-Cre mice (p50-70) with either the channelrhodopsin virus (ChR2, n = 8) or an empty control vector (EV, n = 8) expressed in V1. The mice were head-fixed viewing a static gray screen. Main: Absolute power spectrum (mean +/- SEM) from 100 sec of continuous data during a 'laser on' period. **b.** Aperiodic fit (mean +/- SEM). Inset: Boxplots and FDR-corrected p-values for aperiodic offset (power at 1.5 Hz), aperiodic slope, and knee frequency. For offset, uncorrected p-value = 0.0011 and for knee frequency, uncorrected p-value = 3.1e-4. **c.** Periodic power spectrum (mean +/- SEM). Inset: Pk1 at higher spectral resolution. **d.** Boxplot and p-value for Pk1a center frequency. Dots at bottom of plots in (a-c) indicate the points of significant difference between groups (non-parametric hierarchical bootstrap, 99% confidence interval). Boxplots show 25th, median, and 75th percentiles, with whiskers extending to minimum and maximum values. P-values calculated from two-sample, two-sided Wilcoxon Rank-Sum Tests. Z-statistics and effect sizes not calculated for n < 10. P-values in (b) adjusted with the Benjamini-Hochberg correction. Source data are provided as a Source Data file.

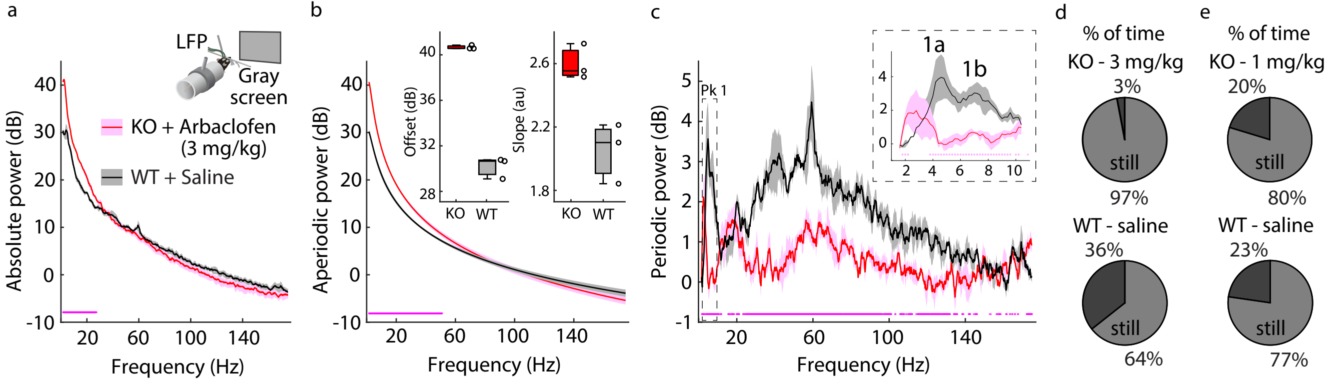
a
LFP
Gray screen
KO + Arbaclofen (3 mg/kg)
WT + Saline
Absolute power (dB)
Frequency (Hz)
b
Aperiodic power (dB)
Offset (dB)
Slope (au)
KO
WT
Frequency (Hz)
c
Pk 1
1a
1b
Periodic power (dB)
Frequency (Hz)
d
% of time
KO - 3 mg/kg
3%
still
97%
WT - saline
36%
still
64%
e
% of time
KO - 1 mg/kg
20%
still
80%
WT - saline
23%
still
77%

Supplementary Figure 11. 3 mg/kg of Arbaclofen suppresses periodic power other than Pk1a and has a sedative effect on *Fmr1*$^{-/y}$ mice. a. Experimental design. V1 L4 LFP data collected in head-fixed juvenile (p30-40) WT and *Fmr1*$^{-/y}$ (KO) mice (n = 3 per group) viewing a static gray screen after systemic injections of saline (for WT) or Arbaclofen (3 mg/kg, for KO). Main: Absolute power spectrum (mean +/- SEM) from 150 sec of data, 60-90 minutes after the injection. **b.** Aperiodic fit (mean +/- SEM). Inset: Boxplot for aperiodic offset (power at 1.5 Hz) and slope. P-values, z-statistics, and effect sizes not calculated for n < 5. **c.** Main: Periodic power spectrum (mean +/- SEM). Inset: periodic Pk1 at higher spectral resolution. **d.** Average percentage of time spent moving and still across 100 sec for the KO mice treated with 3 mg/kg Arbaclofen (top) and the littermate WT mice treated with saline (bottom). **e.** Like (d), but for KO mice treated with 1 mg/kg Arbaclofen (n = 14) compared to saline-treated littermate WT mice (n = 15). Dots at bottom of plots in (a-c) indicate the points of significant difference between groups (non-parametric hierarchical bootstrap, 99% confidence interval). Boxplots show 25th, median, and 75th percentiles, with whiskers extending to minimum and maximum values. Source data are provided as a Source Data file.

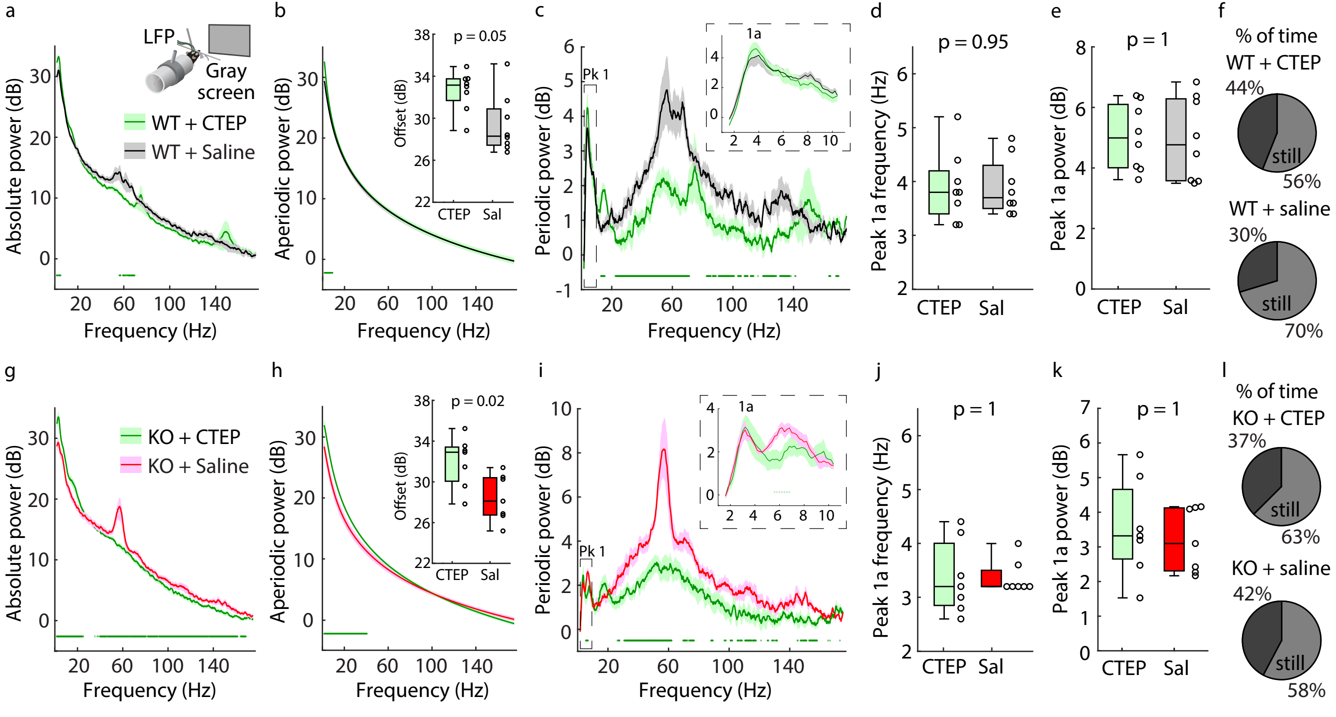
a
LFP
Gray screen
WT + CTEP
WT + Saline
Absolute power (dB)
Frequency (Hz)
b
p = 0.05
Offset (dB)
CTEP
Sal
Aperiodic power (dB)
Frequency (Hz)
c
Pk 1
1a
Periodic power (dB)
Frequency (Hz)
d
p = 0.95
Peak 1a frequency (Hz)
CTEP
Sal
e
p = 1
Peak 1a power (dB)
CTEP
Sal
f
% of time
WT + CTEP
44%
still
56%
WT + saline
30%
still
70%
g
KO + CTEP
KO + Saline
Absolute power (dB)
Frequency (Hz)
h
p = 0.02
Offset (dB)
CTEP
Sal
Aperiodic power (dB)
Frequency (Hz)
i
1a
Pk 1
Periodic power (dB)
Frequency (Hz)
j
p = 1
Peak 1a frequency (Hz)
CTEP
Sal
k
p = 1
Peak 1a power (dB)
CTEP
Sal
l
% of time
KO + CTEP
37%
still
63%
KO + saline
42%
still
58%

Supplementary Figure 12. The mGluR5 NAM CTEP has no effect on periodic Pk1a. a. Inset**:** Experimental design. V1 L4 LFP data collected in head-fixed WT (littermate to *Fmr1*$^{-/y}$) mice (n = 8, p70-90) viewing a static gray screen after systemic injections of saline and 2-chloro-4-[2-[2,5-dimethyl-1-[4-(trifluoromethoxy)phenyl]imidazol-4-yl]ethynyl]pyridine (CTEP, 2 mg/kg). Main: Absolute power spectrum (mean +/- SEM) from 120 sec of data, 90 minutes after each systemic injection. **b.** Main: Aperiodic fit (mean +/- SEM). Inset: Boxplot and p-value for aperiodic offset (power at 1.5 Hz). **c.** Periodic power spectrum (mean +/- SEM). Inset: periodic Pk1 at higher spectral resolution. **d-e.** Boxplot and FDR-corrected p-values for: (d) Pk1a center frequency and (e) Pk1a maximum power (uncorrected p-value = 0.545). **f.** Average percentage of time spent moving and still across 100 sec for all mice after CTEP (top) and after saline (bottom). **g-l.** Like (a-f) but for *Fmr1*$^{-/y}$ mice (n = 7, p70-90, littermates to WT mice in Supplementary Fig. 12a-f) treated with saline (red) or 2 mg/kg CTEP (green). For (k), uncorrected p-value = 0.813. Dots at bottom of plots in (a-c) and (g-i) indicate the points of significant difference between groups (non-parametric hierarchical bootstrap, 99% confidence interval). Boxplots show 25th, median, and 75th percentiles, with whiskers extending to minimum and maximum values. P-values calculated from two-sample, two-sided Wilcoxon Signed-Rank Tests (no multiple comparisons). Z-statistics and effect sizes not calculated for n < 10. Source data are provided as a Source Data file.
