# Supplementary material for: A human electrophysiological signature of Fragile X pathophysiology is shared in V1 of Fmr1-/y mice
Source: Nat Commun. 2026 Feb 9;17:1497. doi: 10.1038/s41467-026-69243-0 (PMC12891484; doi:10.1038/s41467-026-69243-0)
Supplement: Supplementary file 2 — Reporting Summary [file 41467_2026_69243_MOESM2_ESM.pdf]

nature portfolio

Corresponding author(s): Professor Mark F. Bear and Dr Sara S. Kornfeld-Sylla

Last updated by author(s): Jan 15, 2026

Reporting Summary

Nature Portfolio wishes to improve the reproducibility of the work that we publish. This form provides structure for consistency and transparency in reporting. For further information on Nature Portfolio policies, see our Editorial Policies and the Editorial Policy Checklist.

Statistics

For all statistical analyses, confirm that the following items are present in the figure legend, table legend, main text, or Methods section.

n/a	Confirmed	
☐	☒	The exact sample size (*n*) for each experimental group/condition, given as a discrete number and unit of measurement
☐	☒	A statement on whether measurements were taken from distinct samples or whether the same sample was measured repeatedly
☐	☒	The statistical test(s) used AND whether they are one- or two-sided<br>*Only common tests should be described solely by name; describe more complex techniques in the Methods section.*
☐	☒	A description of all covariates tested
☐	☒	A description of any assumptions or corrections, such as tests of normality and adjustment for multiple comparisons
☐	☒	A full description of the statistical parameters including central tendency (e.g. means) or other basic estimates (e.g. regression coefficient) AND variation (e.g. standard deviation) or associated estimates of uncertainty (e.g. confidence intervals)
☐	☒	For null hypothesis testing, the test statistic (e.g. *F*, *t*, *r*) with confidence intervals, effect sizes, degrees of freedom and *P* value noted<br>*Give P values as exact values whenever suitable.*
☒	☐	For Bayesian analysis, information on the choice of priors and Markov chain Monte Carlo settings
☒	☐	For hierarchical and complex designs, identification of the appropriate level for tests and full reporting of outcomes
☐	☒	Estimates of effect sizes (e.g. Cohen's *d*, Pearson's *r*), indicating how they were calculated

Our web collection on statistics for biologists contains articles on many of the points above.

Software and code

Policy information about availability of computer code

Data collection	Human EEG data were collected using either a 128-channel Hydrocel Geodesic Sensor Net (Version 1, EGI Inc, Eugene, OR) connected to a DC-coupled amplifier (Net Amps 300, EGI Inc, Eugene, OR) and NetStation software (version 4.5, EGI Inc, Eugene, OR), or a 128-channel saline Electrical Geodesics system (EGI, MagStim, Minnesota). Mouse EEG data were collected with 1.6mm screw-type electrodes (Protech International) connected to a 4 channel EEG/EMG recording system (Pinnacle Technologies) with the Sirenia Data Acquisition (DAQ) software. Mouse LFP data were collected through tungsten microelectrodes (FHC) connected to either a Plexon 64 Recorder System with a PBX-211 2003 pre-amplifier (Plexon Inc, Dallas, TX, USA) or a Plexon OmniPlex® Neural Recording System, with Plexon's DigiAmp™ acquisition system (Plexon Inc, Dallas, TX, USA), with accompanying Plexon DAQ software. Head-fixed movement data were collected from a piezoelectric disk (C.B. Gitty) placed under the animals' forepaws and acquired concurrently with LFP data in the Plexon DAQ software. Static gray screen visual stimuli for the head-fixed mice with LFP electrodes were generated using previously published, open-source software (https://github.com/jeffgavornik/VEPStimulusSuite) written in either C++ for interaction with a VSG2/2 card (Cambridge Research Systems) or MATLAB (MathWorks, R2012b) using the PsychToolbox extension (version 3.0.12, http://psychtoolbox.org). For optogenetic experiments, light pulses were delivered using a digitally-controlled LED driver (PlexBright® Optogenetic Stimulation System).
Data analysis	Human EEG preprocessing utilized publicly available pipelines run via MATLAB (R2017a or R2021b): Batch Automated Processing Platform (BEAPP) and the Harvard Automated Preprocessing Pipeline for EEG (HAPEE) or the Cincinnati Very High Throughput Pipeline (VHTP) with EEGLAB (2021b). Source localization was conducted with Brainstorm and open M/EEG. Custom code in MATLAB (R2018b) was written for mouse EEG/LFP preprocessing. All further analysis of data from humans and mice, as well as figure generation and statistical analysis, was carried out in MATLAB (R2018b and R2022b). Spectral analysis of human and mouse EEG/LFP was conducted with the publicly available Chronux package (version 2.12), and analyses requiring band-pass filtering utilized the 'eegfilt' function (https://sccn.ucsd.edu/~arno/eeglab/auto/eegfilt.html, copyright 1997). Otherwise, custom code called on built-in MATLAB functions. We have uploaded novel code for analyzing the 1/f signal and temporal dynamics of periodic signals in both EEG and LFP datasets with sample data on Github, available under the

accession code (https://doi.org/10.5281/zenodo.18201252). Our code on Github also includes the custom code written for generation of figures and statistical analyses for this manuscript. We compare the performance of our novel code with previously published code (SpecParam version 1.1.1, https://fooof-tools.github.io/fooof/, implemented in MARLAB R2018b under the MATLAB wrapper https://github.com/fooof-tools/fooof_mat) to describe the regions of the spectrum where the fit is improved for our code.

For manuscripts utilizing custom algorithms or software that are central to the research but not yet described in published literature, software must be made available to editors and reviewers. We strongly encourage code deposition in a community repository (e.g. GitHub). See the Nature Portfolio guidelines for submitting code & software for further information.

Data

Policy information about availability of data

All manuscripts must include a data availability statement. This statement should provide the following information, where applicable:

- Accession codes, unique identifiers, or web links for publicly available datasets
- A description of any restrictions on data availability
- For clinical datasets or third party data, please ensure that the statement adheres to our policy

Preprocessed resting-state data from mice used to produce the figures in this manuscript are available on Figshare under the accession code (https://doi.org/10.6084/m9.figshare.29940884). Resting-state data are only a portion of the raw Plexon and Pinnacle murine data files, which available upon request from the authors. Please contact the corresponding authors with such requests; the expected timeframe for response is one week. Source data are provided with this paper.

For data from human subjects, consents obtained from participants prohibit sharing of identifiable or de-identified individual data without data use agreements in place. The current study includes data collected from several independent projects, including some in which consent for open sharing was not collected from the subjects. Thus, our ability to share data from human subjects depends on a variety of factors: which specific data are requested; whether and to what extent the participants included in the requested data have consented to the sharing and future use of their data; whether deidentified, limited, or identified data are requested; and the purpose for which the data are requested. Prior to sharing data, we may need to confirm whether Cincinnati Children's Hospital and Medical Center or Boston Children’s Hospital and the institution of the individual requesting the data have existing agreements or subcontracts with terms of data use and sharing that define the collaboration (e.g., data use agreements, data access agreements, subawards, reliance agreements). Please contact the corresponding authors with data requests to determine availability of the specific data of interest. If data use or data access agreements are needed, approval between institutions can take at least 2-3 months. Once approved, data is shared for the length of the agreement (usually 1 year, with opportunity for renewal).

Research involving human participants, their data, or biological material

Policy information about studies with human participants or human data. See also policy information about sex, gender (identity/presentation), and sexual orientation and race, ethnicity and racism.

Reporting on sex and gender	This study uses the term sex to describe the sex assigned at birth. For data from children, parents were asked their child's sex using a parent survey as part of the study. For data from adults, sex was determined by self-report for typically-developing subjects and by caregiver report for subjects with FXS. For both children and adults, sex for subjects with FXS was corroborated by the genetic testing done to confirm the full mutation of FMR1.<br><br>Our manuscript focused on male subjects. The human FMR1 and mouse Fmr1 genes are on the X-chromosome, FXS diagnoses are most prevalent in males, and the electrophysiological phenotypes are most prominent in the male subjects. The goal of this work was to establish a parallel disruption of alpha oscillations in FXS across species, so we focused on male humans and mice to this end. While female humans with FXS also show this phenotype to a lesser extent, there are sexually dimorphic phenotypes in FXS, including in alpha oscillations. Therefore, we hope that future work will investigate if there are parallel alterations in alpha oscillations in human females with FXS and in female FXS model mice.
Reporting on race, ethnicity, or other socially relevant groupings	Race and ethnicity are not reported in this manuscript. Race and ethnicity information were optionally requested either from the parent of the participant when completing a demographics survey (for children), from the participant themselves through self-report (for typically-developing adults), or from caregivers (for FXS adults). However, not everyone responded, so we did not analyze this as a covariate.
Population characteristics	In the first two studies with children, data were collected from 17 males (27-78 months old) with full mutation of FMR1 and 17 age-matched (27-80 months) typically-developing males. In the study with adults, data were collected from 20 males (19-43 years old), 20 with a full mutation of FMR1 and 20 age-matched (20-44 years old) typically-developed males. Age was the only covariate studied.
Recruitment	For the study with children, TD participants were recruited through a hospital research registry as well as social media advertisement. FXS participants were recruited directly from the hospital FXS clinic, social media advertisement, and through email sent to families in Fragile X societies and groups. Bias can exist in who is able to participate in research, as families must be aware of the study to participate, and must have the time and resources to be able to come to multiple 3-4 hour study visits. To reduce this bias, additional funding was available for FXS families to help cover travel costs from other states.<br><br>For the study with adults, TD participants were recruited through hospital-wide social media and email/flyers posts. Participants with FXS were primarily recruited through the Cincinnati Fragile X Research and Treatment Center. In addition, FXS participants were recruited at the bi-annual international Fragile X conference as well as through recruitment posts from the National Fragile X Foundation. Self-selection bias was unlikely but it is possible that certain FXS families declined to inquire about the study due to concern their child could not complete (this is unlikely for the TD participants).
Ethics oversight	Approval was obtained prior to starting each study from the Institutional Review Board at Boston Children’s Hospital or the Institutional Review Board at Cincinnati Children’s Hospital and Medical Center, depending on the site where the study was conducted. Written, informed consent was obtained from either the participant or from the participant's parent/guardian

with assent from the participant. For the pediatric study, written, informed consent was obtained from all parents or guardians prior to their children's participation in the study.

Note that full information on the approval of the study protocol must also be provided in the manuscript.

Field-specific reporting

Please select the one below that is the best fit for your research. If you are not sure, read the appropriate sections before making your selection.

- [x] Life sciences
- [ ] Behavioural & social sciences
- [ ] Ecological, evolutionary & environmental sciences

For a reference copy of the document with all sections, see nature.com/documents/nr-reporting-summary-flat.pdf

Life sciences study design

All studies must disclose on these points even when the disclosure is negative.

Sample size

No sample size calculations were performed. For humans, the final sample was limited by recruitment of a rare genetic disorder as well as successful high quality EEG collection in children with a neurodevelopmental disorder. Prior studies in FXS had used a smaller sample size (n=11) and found significant differences in resting state EEG measures. Thus data available from a larger sample of FXS children (n=17) was used. A comparable sample size in adult subjects was therefore used (n = 20), randomly selected from unpublished portions of large datasets collected across two federally-funded studies (NIH U54HD082008 and U54HD104461). For mice, resting state LFP measurements had been collected in the lab as baseline measurements in various experiments dating back 10+ years, and all available resting-state data from naive (i.e., before the experiments began) Fmr1 KO and littermate control mice were analyzed for this paper. For the optogenetic experiments in SOM-Cre mice, the sample size used was consistent with previous studies using optogenetics or chemogenetics to manipulate SOM+ activity and measure resulting signals in V1 (see Huang et al, 2020). Similarly, for Arbaclofen experiments, the sample size used was consistent with previous studies testing the effects of Arbaclofen or Racemic Baclofen on the EEG of Fmr1 KO mice (see Janz et al, 2025 and Jonak et al, 2022).

Data exclusions

For the study with human children, participants with FXS were excluded if they did not have a full mutation of FMR1, or if any participant had a history of prematurity (<35 weeks gestational age), low birth weight (<2000gms), known birth trauma, known genetic disorders (other than FXS), unstable seizure disorder, current use of anticonvulsant medication, and uncorrected hearing or vision problems. 2-second EEG segments with retained artifact after ICA/MARA were rejected using HAPPE's amplitude and joint probability criteria. EEG were rejected for data quality if they had fewer than 10 segments (20 seconds total) or did not meet the following HAPPE data quality output parameters: percent good channels >80%, mean and median retained artifact probability <0.3, percent of independent components rejected <80%, and percent variance after artifact removal <32%.

For the study with human adults, participants with FXS were excluded if they did not have a full mutation of FMR1, if they had other known syndromic conditions associated with intellectual disability (other than FXS; e.g., Down Syndrome), if they were on benzodiazepines or anticonvulsant medications, or if they were on any novel potential treatment (e.g., minocycline) known to affect EEG measures within 4 weeks of testing. Typically developed adult controls were excluded if they scored ≥ 8 on the Social Communication Questionnaire, if they had a history of psychiatric or neurologic disorders, or had a first or second-degree relative with autism spectrum disorder or a serious psychiatric illness. Sections of poor quality EEG data were manually rejected, and 2-second EEG segments with retained artifact exceeding +/-120 µV after ICA were automatically rejected using an automatic amplitude rejection threshold.

For mice, animals were excluded if they had a poor LFP implant. This was the exclusion criteria for both LFP and EEG datasets, as the mice with EEG also had LFP implants in the same hemisphere. An LFP implant was considered unusable if the power spectrum had too much noise to quantify periodic peaks, if the power below 60 Hz fell below 0 dB, and/or if the magnitude of the average visually-evoked potential for a grating stimulus was less than 40 microvolts. Very few animals (< 10 out of 300) were excluded according to this criteria. For mice that had concurrent forepaw movement measured with a piezoelectric disk, a small number of 5-second epochs within the 50-150 second time-series data were excluded from analysis due to occasional contamination of the piezo signal into the LFP signal during certain movement bouts. The very small fraction of epochs (usually 0-2 per mouse) were eliminated if they contained both a large movement artifact and a huge spike in low frequency power (less than 1.5 Hz). All remaining epochs were included in analysis. For the microburst analysis, mice were excluded if they did not have at least 100 seconds of continuous data free of large motion artifacts that spiked low frequency power.

Replication

Human adult data replicate and extend previous findings (see citations in main text). For data from children, the results were consistent across two studies which were combined for this manuscript. For Fmr1 KO mice, the results were reproducible and consistent across many different experiments and six experimentalists over a span of 10 years (hence the large sample sizes). Moreover, the elevated gamma power observed in all Fmr1 KO mice (and the reduction of the phenotype by Arbaclofen) replicates and extend previous findings (see citations in main text). For optogenetic experiments, the data are consistent with the effects of optogenetically manipulating SOM+ cells on visually-evoked theta oscillations in mouse V1 (see Huang et al, 2020). Therefore, all attempts at replication in this study were successful.

Randomization

Humans were not randomized in the study, as groups were determined by genetic background and/or diagnostic characterization and there was no additional experimental/treatment condition. For mice that received a viral injection and/or drug treatment (including opto/chemogenetic experiments), treatment was randomized by cage so littermate pairs received matching treatments.

Blinding

For human data, experimenters could not be blinded because of the behavioral differences between subject groups but preprocessing of the EEG data was either blinded (for adults) or fully automated and reproducible (for children). For mice, the experimenter was blinded to genotype during surgery, drug treatment (if applicable), and data collection. Subsequent data analysis of EEG and LFP signals was automated. For chemogenetic experiments, the experimenter was blinded to treatment (see Kaplan et al, 2016). For drug experiments using CTEP, the experiment was also blinded to treatment due to a crossover design. A crossover design was not used for Arbaclofen experiments in order to collect both treatment conditions within the same day.

Reporting for specific materials, systems and methods

We require information from authors about some types of materials, experimental systems and methods used in many studies. Here, indicate whether each material, system or method listed is relevant to your study. If you are not sure if a list item applies to your research, read the appropriate section before selecting a response.

Materials & experimental systems

n/a	Involved in the study
☒	☐ Antibodies
☒	☐ Eukaryotic cell lines
☒	☐ Palaeontology and archaeology
☐	☒ Animals and other organisms
☒	☐ Clinical data
☒	☐ Dual use research of concern
☒	☐ Plants

Methods

n/a	Involved in the study
☒	☐ ChIP-seq
☒	☐ Flow cytometry
☒	☐ MRI-based neuroimaging

Animals and other research organisms

Policy information about studies involving animals; ARRIVE guidelines recommended for reporting animal research, and Sex and Gender in Research

Laboratory animals	Fmr1 KO mice were obtained from Jackson Laboratories, Maine, USA (stock # 003025) and backcrossed onto a C57BL/6J background for at least six generations at Massachusetts Institute of Technology or King's College London. Fmr1 cKO mice were also obtained from Jackson Labs (stock # 035184) and crossed with Emx1-Cre mice (Jackson stock # 005628). As previously reported, for hM4D(Gi) experiments, mice were Parvalbumin-Cre recombinase knock-in mice (B6;129P2-Pvalbtm1(cre)Arbr/J, PV-Cre) on a C57BL/6 background (Jackson stock # 017320). For optogenetic inhibition experiments, SOM-Cre mice were used (B6J.Cg-Ssttm2.1(cre)Zjh/MwarJ, Jackson stock # 028864). Experimental cohorts consisted of male littermates that were weaned at p21 and were p30-P150 at the time of experiments Experimental cohorts consisted of male littermates that were weaned at p21 and were p30-P150 at the time of experiments (n = 215 Fmr1-/y mice and littermate controls, p30-40 and p70-150, n = 44 Emx1-Fmr1 KO mice and littermate controls, p30-40, n = 16 PV-Cre mice, p50-80, and n = 31 Som-Cre mice and littermate controls, p50-70). Mice were maintained on a 12-hour light-dark cycle (7am – 7pm) in a temperature- and humidity-controlled animal care facility (68-72° F and 30-70% humidity) with ad lib access to food and water and housed with 1-4 other littermates. All experiments were performed blind to genotype using age-matched littermate controls during the light phase.
Wild animals	No wild animals were used
Reporting on sex	Male mice were used in this study (see reporting on Sex and Gender for human participants for the justification for both species).
Field-collected samples	No field-collected samples were involved in this study
Ethics oversight	All experimental techniques were approved by The Institutional Animal Care and Use Committees and Department of Comparative Medicine at MIT (Bear protocol: 2403000639) and conformed to the Guide for the Care and Use of Laboratory Animals published by the National Institutes of Health, or were approved by the Ethical Committee for Animal Use of King's College London (Home Office License: PF82DF031-5) and were in accordance with the United Kingdom Home Office Animals (Scientific Procedures) Act 1986, depending on the site where the data were collected.

Note that full information on the approval of the study protocol must also be provided in the manuscript.

Plants

Seed stocks	*Report on the source of all seed stocks or other plant material used. If applicable, state the seed stock centre and catalogue number. If plant specimens were collected from the field, describe the collection location, date and sampling procedures.*
Novel plant genotypes	*Describe the methods by which all novel plant genotypes were produced. This includes those generated by transgenic approaches, gene editing, chemical/radiation-based mutagenesis and hybridization. For transgenic lines, describe the transformation method, the number of independent lines analyzed and the generation upon which experiments were performed. For gene-edited lines, describe the editor used, the endogenous sequence targeted for editing, the targeting guide RNA sequence (if applicable) and how the editor was applied.*
Authentication	*Describe any authentication procedures for each seed stock used or novel genotype generated. Describe any experiments used to assess the effect of a mutation and, where applicable, how potential secondary effects (e.g. second site T-DNA insertions, mosiacism, off-target gene editing) were examined.*
